# An Inhibitor of NF-κB and an Agonist of AMPK: Network Prediction and Multi-Omics Integration to Derive Signaling Pathways for Acteoside Against Alzheimer’s Disease

**DOI:** 10.3389/fcell.2021.652310

**Published:** 2021-07-19

**Authors:** Ying-Qi Li, Yi Chen, Si-Qi Jiang, Yuan-Yuan Shi, Xiao-Li Jiang, Shan-Shan Wu, Ping Zhou, Hui-Ying Wang, Ping Li, Fei Li

**Affiliations:** ^1^State Key Laboratory of Natural Medicines, China Pharmaceutical University, Nanjing, China; ^2^College of Pharmacy, Xinjiang Medical University, Urumqi, China

**Keywords:** acteoside, BV-2 cells, metabolism, RNA-seq, mitochondria, neuroinflammation

## Abstract

Alzheimer’s disease (AD) is the most frequent type of dementia. Acteoside (ACT) is a compound isolated from *Cistanche tubulosa*, which possesses excellent neuroprotective properties. However, the underlying mechanism of ACT in regulating microglia polarization remains ill-defined. Therefore, a computational network model was established to identify the driving targets of ACT and predict its mechanism by integrating multiple available databases. The AlCl_3_-induced AD model in zebrafish larvae was successfully constituted to demonstrate the therapeutic efficacy of ACT. Subsequently, LPS-induced BV-2 cells uncovered the positive role of ACT in M1/M2 polarization. The NF-κB and AMPK pathways were further confirmed by transcriptomic analysis, metabolomics analysis, molecular biology techniques, and molecular docking. The research provided an infusive mechanism of ACT and revealed the connection between metabolism and microglia polarization from the perspective of mitochondrial function. More importantly, it provided a systematic and comprehensive approach for the discovery of drug targets, including the changes in genes, metabolites, and proteins.

## Introduction

With the aging and rapid growth of population, the number of dementia cases in the world has shown an upward trend. Alzheimer’s disease (AD) is a common neurodegenerative disease accompanied by cognitive impairment and dyskinesia (Perea et al., 2020), which is the most frequent type of dementia. It is characterized by severe neuronal loss, senile plaques, and neurofibrillary tangles (Shiao et al., 2017). The pathogenesis of AD is multidimensional and links to neuroinflammation. Neuroinflammation is driven by the activation of glial cells, which is closely related to the occurrence and development of AD (Hanslik and Ulland, 2020; Linnerbauer and Rothhammer, 2020). During the progression and exacerbation of neuroinflammation, microglia is considered as a key factor.

Microglia are the primary immune cells in the central nervous system, which are closely associated with a cascade of processes such as brain development, maintenance of neural environment, as well as the responses to injury and repair (Perea et al., 2020). Moreover, microglia can be stimulated to an M1 phenotype and increase the expression of pro-inflammatory cytokines when neuroinflammation-related diseases such as AD occurs (Tsukahara et al., 2020). Studies also demonstrate that the polarization to M1 phenotype is often accompanied by metabolic disorders (Li L. et al., 2020), leading to energy metabolism imbalance and mitochondrial dysfunction (Agrawal and Jha, 2020). These adverse changes are derived to neurodegeneration, even AD.

Acteoside (ACT), a phenylethanoid glycoside, is primarily derived from *Cistanche tubulosa*. Increasing evidence has suggested that ACT possesses numerous pharmacological activities, including neuroprotective (Wei et al., 2019), anti-inflammatory (Lai et al., 2019), and antioxidant (Ji et al., 2019) effects. In particular, it has been proven that ACT can improve learning and memory impairment as well as upregulate energy metabolism in streptozotocin-induced rats (Chen J. et al., 2020). In addition, it can also inhibit neuronal apoptotic cell death and mitochondrial damage in the experimental autoimmune encephalomyelitis mice (Li W. et al., 2020). However, the effect of ACT on microglia M1/M2 polarization and its mechanism are rarely reported. Up to now, the mechanisms such as the repair of mitochondria function and the regulation of cell metabolism are unclear and further scientific research is needed to clarify the exact mechanism.

The purpose of the present report is to investigate the therapeutic efficacy of ACT as well as the underlying molecular mechanism of ACT on AD. Herein, a systematic *in silico* method of drug target identification is established based on the consolidated databases. We further investigate the possible mechanisms of ACT at the molecular biological level by integrating RNA-sequencing (RNA-seq) with metabolomics method and molecular biology techniques. The combination of *in silico*, *in vivo*, and *in vitro* systematic screening strategy provides a new protocol to objectively discover multi-target compounds of traditional Chinese medicine.

## Materials and Methods

### Network Modeling

Four online platforms were combined to predict and discover the targets of ACT, namely, GeneCards^1^, similarity ensemble approach (SEA^2^), SwissTargetPrediction^3^, and PharmMapper^4^. All gene associations of AD were independently collected from DisGeNET^5^ and GeneCards. After the target–target interaction analysis conducted by String database^6^, the network was further integrated by Cytoscape (Version 3.8.2) software. GO and KEGG enrichment analysis were performed on the DAVID database^7^ to annotate and mine the network.

### Animals and Model Grouping

Wild-type zebrafish (AB strain, 4 months old) were chosen in this study (Nanjing Qi Wu Biotechnology Co., Ltd.). They were maintained under a 14/10-h light/dark cycle at 28°C, following the previous method (Li Y. Q. et al., 2020). All zebrafish experiments were carried out under the supervision of the Animal Ethics Committee of China Pharmaceutical University.

Zebrafish larvae were divided into six groups and treated from 3 days post-fertilization (dpf) to 7 dpf: control group, model group, model + donepezil hydrochloride (DPZ, Shanghai Aladdin Bio-Chem Technology Co., Ltd.) group, and model + ACT (HPLC purity ≥ 98%, Baoji Herbest Bio-Tech Co., Ltd.) groups. The control group was maintained in the medium with 0.2% DMSO and the model group was treated with 150 μM AlCl_3_ (pH 5.8). The model + DPZ group was co-treated with AlCl_3_ and 8 μM DPZ. The model + ACT groups were co-treated with AlCl_3_ and different concentrations of ACT (200, 100, and 50 μM).

### Behavioral Analysis

Zebrafish larvae movements were recorded by a ViewPoint behavioral analyzer (Zebralab, 2018, ViewPoint Life Sciences Co., Ltd.) at 28°C. Briefly, the behavioral parameters and result processing were consistent with the method we established earlier (Li Y. Q. et al., 2020). Herein, average speed (AS), speed change (ΔS), dyskinesia recovery rate (DRR), and response efficiency (RE, %) were selected to evaluate dyskinesia recovery in zebrafish.

### Determination of Acetylcholinesterase and Choline Acetyltransferase Activity

After treating from 3 to 7 dpf, zebrafish larvae were collected to measure Acetylcholinesterase (AChE) and Choline Acetyltransferase (ChAT) activities. Based on the manufacturer’s protocol, the activity was detected by the enzyme-linked immunosorbent assay (ELISA) kits (MLBIO Biotechnology Co., Ltd., Shanghai, China).

### Cell Cultures and Treatments

BV-2 cell line was purchased from the American Type Culture Collection (ATCC, Manassas, VA, United States). They were cultured in DMEM (KeyGen Biotech Co., Ltd., Nanjing, China). The medium was supplemented with 10% FBS (Gibco, Grand Island, NY, United States), 100 U/ml penicillin, and 100 mg/ml streptomycin, accompanied with 95% air/5% CO_2_ at 37°C. BV-2 cells were incubated with ACT (50, 25, and 12.5 μM) or stimulated with lipopolysaccharide (LPS, 1 μg/ml; Sigma-Aldrich, St Louis, MO, United States) for 24 h. The cells were observed under the inverted microscope (Nikon ECLIPSE Ti2, Japan).

### Cell Viability Assay

Cell Counting Kit-8 (CCK-8) assay (JianCheng Bioengineering Institute, Nanjing, China) was used to evaluate the cell viability. Briefly, the absorbance was measured at 450 nm with a microplate reader (Bio-Tek Instrument, Winooski, VT, United States).

### Nitric Oxide Production Assay

Nitric oxide (NO) was determined by measuring nitrite levels in the BV-2 culture supernatant using Griess reagent. Briefly, the medium (100 μl) was transferred to a new 96-well plate, the same volume of Griess reagent was added to each well and reacted for 15 min in the dark. The absorption at 540 nm was determined by a Microplate Reader.

### Inflammatory Cytokines Levels in Supernatant

The concentrations of TNF-α, IL-1β, and IL-10 in the BV-2 cell supernatant were determined by ELISA kits according to the manufacturer’s instructions.

### Cellular Metabolism Determination by HPLC-Q-TOF-MS Analysis

BV-2 cells were seeded in a six-well dish separately (*n* = 6/group). After treatment, the medium was removed, and the cells were washed three times with cold PBS. They were then immediately exposed to liquid nitrogen to suppress cell metabolism. The cells were harvested with 80% cold methanol. To facilitate protein precipitation, cells were vigorously vortexed for 1 min and centrifuged at 13,000 rpm (15 min, 4°C). The cell suspension was dried under a stream of nitrogen. The dried residue was reconstituted in 150 μl of pre-cooled 25% acetonitrile. In order to ensure the stability and accuracy of the sequence analysis, each cell sample with equal volume (10 μl) was combined as quality control samples. During metabolite detection, these samples were injected after every six samples to confirm their stability. A 1-μl aliquot was injected for HPLC-Q-TOF-MS.

The analysis was performed on an Agilent 1290 HPLC system connected with the Agilent 6530 Quadrupole Time-of-Flight (Q-TOF) mass spectrometer (Agilent Technologies, Santa Clara, CA, United States). The separation was carried out on an ACQUITY UPLC BEH C_18_ column (2.1 min × 100 mm, 1.7 μm). The mobile phase was composed of 0.1% formic acid–water (v/v; A) and acetonitrile (B).

The flow rate was set at 0.4 ml/min with the following optimal gradient elution condition: 0 to 2 min, 5% B; 2 to 20 min, 5 to 95% B (positive ion mode); 0 to 2 min, 5% B; 2 to 20 min, 5 to 95% B (negative ion mode). The operation parameters of the mass spectrometer were set as follows: gas temperature, 320°C; drying gas, 10 L/min; nebulizer, 35 psi; VCap, 4,000 V; fragmentor, 120 V.

The raw data were operated under MassHunter Workstation Software version B.07.00 (Agilent Technologies, Santa Clara, CA, United States). The raw data were pre-processed by the XCMS platform. Principal components analysis (PCA) and partial least-squares discriminant analysis (PLS-DA) of the normalized data were conducted with MetaboAnalyst^8^. Combined with the literature, the differential metabolites (VIP > 1, *t*-test *p* < 0.05) were identified on HMDB^9^. Finally, pathway analysis was conducted with MetaboAnalyst.

### Measurement of Mitochondrial Membrane Potential

Mitochondrial membrane potential (MMP) was detected using fluorescent probe JC-1 (Beyotime, China) in accordance with the manufacturer’s instructions. Briefly, cells from different groups were rinsed with PBS and incubated with JC-1 staining solution for 20 min at 37°C. After staining, cells were washed twice using staining buffer. Then, fluorescent signals were detected by flow cytometry (BD Accuri C6).

### Measurement of Mitochondrial Adenosine 5′-Triphosphate

Adenosine 5′-triphosphate (ATP) concentration in mitochondria was detected by an ATP Assay Kit (Beyotime, China). Briefly, the culture medium of BV-2 cells from different groups was discarded, and cells were homogenized with lysis buffer on ice. The supernatant obtained after centrifugation (12,000 *g*, 5 min) was used to determine the ATP concentration. The luminescence was detected by EnVision Multimode Microplate Reader (PerkinElmer).

### Measurement of Intracellular Reactive Oxygen Species Level

Reactive oxygen species (ROS) Assay Kit (Beyotime, China) was used to measure ROS level. The cells from different groups were incubated with DCFH-DA (10 μM) for 20 min at 37°C. After probe loading, cells were washed three times with DMEM. Then, fluorescent signals were detected by flow cytometry (BD Accuri C6).

### Transmission Electron Microscopy

BV-2 cells were seeded in a six-well dish. The medium was removed and 1 ml of 2.5% glutaraldehyde was rapidly added to each well. Then, the cells were gathered and fixed with new 2.5% glutaraldehyde at 4°C overnight. After fixation, dehydration, and embedding, the cells were observed with an HT7800 transmission electron microscope (Hitachi, Tokyo, Japan).

### RNA-Seq and Bioinformatic Data Analysis

Total RNA from BV-2 cells were extracted using Trizol reagent (Vazyme Biotech, China). All analytical samples were sent to Majorbio (Shanghai Majorbio Bio-pharm Technology Co., Ltd.) for the RNA sequence assay. The data were analyzed on the online platform of Majorbio Cloud Platform^10^. RSEM^11^ was used to quantify gene abundances. Essentially, differential expression analysis was performed using the DESeq2/DEGseq/EdgeR with *Q* value ≤ 0.05; DEGs with | log2FC| > 1 and *Q* value ≤ 0.05 (DESeq2 or EdgeR)/*Q* value ≤ 0.001 (DEGseq) were considered to be significantly different expressed genes. In addition, functional-enrichment analysis including GO and KEGG were performed to identify which DEGs were significantly enriched in GO terms and pathways at Bonferroni-corrected *p*-value ≤ 0.05 compared with the whole-transcriptome background. The original sequence data have been submitted to the database of the NCBI Sequence Read Archive.

### Quantitative Real-Time Polymerase Chain Reaction

The total RNA of BV-2 cells was harvested using RNA-easy^TM^ Isolation Reagent (Vazyme Biotech, China), and reverse transcription reaction was conducted with FastKing-RT SuperMix (TIANGEN Biotech, China). Reactions were performed according to the manufacturer’s protocol. cDNA was subjected to quantitative real-time polymerase chain reaction (qRT-PCR) assays with specific primers and TransStart TOP Green qPCR SuperMix (TransGen Biotech, China). The primers are listed in Supplementary Table 1 and β-actin was used as the internal control. The 2^–Δ^ ^Δ^ ^*CT*^ method was used for quantitative analysis.

### Western Blot Analysis

BV-2 cells were lysed by RIPA lysis buffer (KeyGen Biotech Co., Ltd., Nanjing, China) containing 1% Protease Inhibitor Cocktail (Thermo Fisher) to obtain total protein. The proteins were separated by 10% SDS-PAGE and transferred to NC membranes. After blocking with 5% skimmed milk/BSA, the membranes were incubated with AMPKα (Proteintech), p-AMPKα (Affinity Biosciences), PGC-1α (Proteintech), NF-κB (Proteintech), p-NF-κB (ABclonal), or GAPDH (ABclonal) antibodies in 5% TBST at 4°C overnight. The membranes were incubated with a secondary horseradish peroxidase-conjugated antibody (ABclonal) at room temperature for 1 h. The high-sig ECL Western blotting substrate (Tanon, China), Gel imaging system (Tanon, China) and ImageJ software were used for visualization and quantitation.

### Molecular Docking

Molecular docking analysis was performed using Autodock software (Version 4.2). The affinity between ACT and proteins were observed by AutodockTools software. The three-dimensional (3D) protein structures of AMPKα (PDB ID: 5g5j) and NF-κB (PDB ID: 4q3j) were retrieved from the Protein Data Bank^12^.

### Statistical Analysis

All data are expressed as the mean ± standard deviation and analyzed by GraphPad Prism Software (Version 8.0.1). The differences between the groups were analyzed by one-way analysis of variance (ANOVA), followed by Tukey’s multiple comparison test. *p* < 0.05 was considered statistically significant.

## Results

### ACT-AD Targets Interaction Network and Pathway Prediction

**FIGURE 1:**
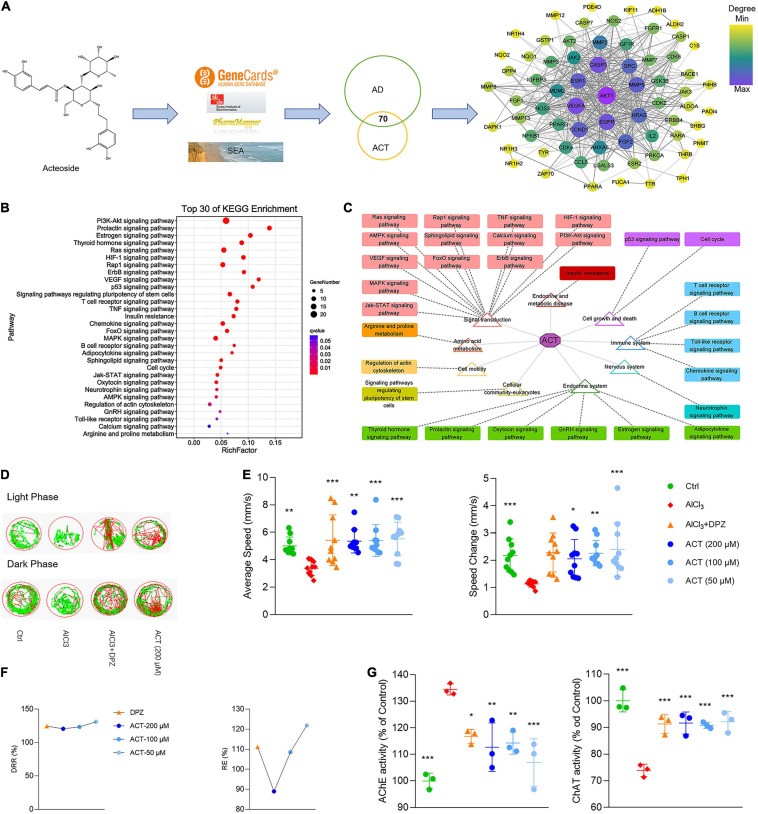
ACT attenuated AlCl_3_-induced AD in zebrafish larvae. **(A)** The workflow of ACT-AD targets interaction network modeling. The size and color of nodes represented the degree of targets. The width of edges was consistent with the combined score of targets. **(B)** Top 30 of KEGG enrichment. **(C)** Illustration of KEGG pathway enrichment analysis. **(D)** The representative swimming trails of zebrafish larvae in the light and dark phase. **(E)** The average speed and speed change in different groups (*n* = 10). **(F)** DRR (%) and RE (%) in the drug treatment groups (*n* = 10). **(G)** The evaluation of AChE and ChAT activities (*n* = 3). *P < 0.05, **P < 0.01, and ***P < 0.005, versus the AlCl_3_ group. DPZ as the positive control.

Through the consolidation of multiple databases, 253 targets of ACT were acquired, whereas a total of 1,697 targets related to AD were gathered. After target network mapping, a total of 70 targets were absorbed into the ACT-AD target interaction network (Figure 1A). KEGG enrichment analysis implied that ACT could have a broad-spectrum and multiple target effect (Figure 1B). It also suggested that the ACT has multiple pathways, target points and elements in AD treatment. Classify these pathways to obtain accurate interpretation of the mechanisms. ACT might mainly correlate with signal transduction, the endocrine system, the immune system, and cell growth and death to play a confrontational role against AD (Figure 1C). It is worth noting that the vast majority of these pathways are related to inflammation reaction. It is speculated that the anti-inflammatory action of ACT might be an inextricable part of its confrontational role against AD.

### ACT Alleviated Dyskinesia and Improved the Cholinergic System Function in AD Zebrafish Larvae

To verify the network model, the AlCl_3_-induced AD model in zebrafish larvae was used to demonstrate the effect of ACT. The zebrafish larvae movement within light/dark cycles was observed, and their swimming trails were recorded (Figure 1D). The results showed that ACT effectively increased the AS and ΔS of zebrafish, and the synergistic effect increased with the increase of doses (Figure 1E). DRR and RE uncovered a more intuitive comparison of ACT and DPZ (Figure 1F). Accordingly, ACT alleviated dyskinesia, exhibiting similar effects to DPZ.

It is generally agreed that the cholinergic system plays an important role in learning and memory processes. Thus, the activities of AChE and ChAT were used to reveal the effect of ACT. AlCl_3_ exposure in zebrafish was rendered with brain cholinergic alteration (Figure 1G). It was significant that ACT treatment suppressed the activity of AChE. In addition, the activity of ChAT exhibited a decrease after ACT treatment. Therefore, ACT showed a profound impact in AlCl_3_-induced AD zebrafish larvae.

### ACT Suppressed M1 Polarization and Promoted M2 Polarization in LPS-Induced BV-2 Cells

**FIGURE 2:**
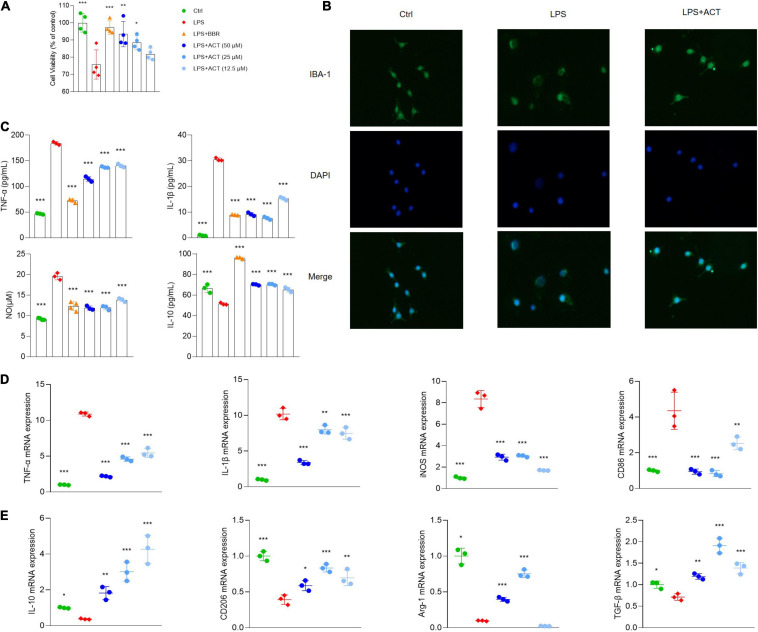
ACT regulated M1/M2 polarization in LPS-stimulated BV-2 cells. **(A)** Effects of ACT on LPS-induced BV-2 cell viability. **(B)** Cell morphology with IBA-1 staining was observed with inverted phase contrast fluorescence microscope (×10). The control group exhibited small soma with distal arborization, showing the typical ramified morphology of resting microglia. The LPS group became fewer and shorter branches with a greatly enlarged cell body, the characteristic shapes of activated microglia. The ACT groups showed attenuated LPS-induced morphological changes. **(C)** Effects of ACT on LPS-induced elevation of cell supernatant TNF-α, IL-1β, and IL-10 levels as well as NO elevation. *P < 0.05, ***P < 0.005, versus the LPS group. *n* = 3. Berberine (BBR) as a positive control. **(D)** Effects of ACT on M1 microglia polarization related markers. **(E)** Effect of ACT on M2 microglia polarization related markers. *P < 0.05, **P < 0.01, ***P < 0.005, versus the LPS group. *n* = 3.

To further confirm the network, the anti-inflammatory effect of ACT was studied *in vitro* using BV-2 microglial cells. After LPS treatment for 24 h, the viability of BV-2 cells decreased significantly. Fortunately, ACT increased the cell viability of LPS-induced BV-2 cells (Figure 2A). In addition, the morphology of BV-2 cells was observed. After 24 h of LPS stimulation, it showed that BV-2 cells underwent a M1 polarization state. The morphological changes were prevented by ACT co-treatment (Figure 2B).

Moreover, the results indicated that, unlike BV-2 cells stimulated by LPS, BV-2 cells co-treated with ACT significantly suppressed TNF-α, IL-1β, and NO (Figure 2C) expressions in cell supernatant. These are classical pro-inflammatory cytokines as indicators of M1 microglia polarization. Similar to the results of ELISA, the results of qPCR discovered that TNF-α, nitric oxide synthase (iNOS), IL-1β, and CD86 mRNA expressions were significantly inhibited by ACT treatment compared with the LPS group (Figure 2D).

Furthermore, we measured M2 microglia polarization level by ELISA (Figure 2C) and qPCR (Figure 2E), and the results indicated that ACT significantly increased M2 microglia-related marker expression levels (IL-10, CD206, TGF-β, and Arg-1). In a word, these results showed that ACT suppressed M1 microglia polarization and promoted the M2 phenotype.

### ACT Regulated M1/M2 Polarization *via* the Inhibition of the NF-κB Pathway

**FIGURE 3:**
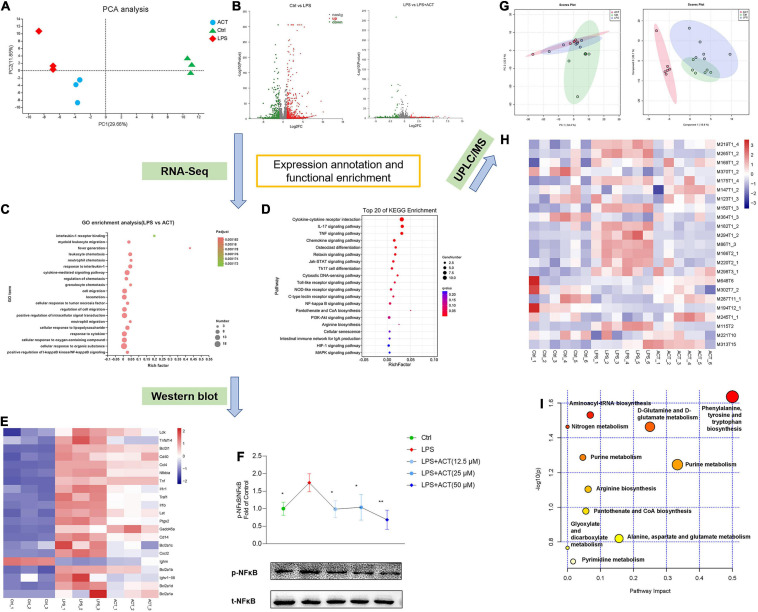
ACT regulated M1/M2 polarization *via* the inhibition of NF-κB signaling pathway and positively regulated the metabolism shifts in LPS-induced BV-2 cells. **(A)** Principal components analysis (PCA) score plot for discriminating the cell metabolome from Ctrl, LPS, and ACT (50 μM) groups from a transcriptomic level (*n* = 3). **(B)** Volcano plot representing the RNA-seq data of LPS-induced and ACT-treated BV-2 cells. **(C)** GO terms in ACT-treated cells as determined by GO enrichment analysis. **(D)** Top 20 pathways in ACT-treated cells as determined by KEGG enrichment analysis. **(E)** Heat map of the differentially expressed genes (DEGs) that were linked to the NF-κB signaling pathway. **(F)** ACT distinctly decreased the phosphorylation level of NF-κB (*n* = 3). *P < 0.05, **P < 0.01, versus the LPS group. **(G)** PCA score plot and partial least squares-discrimination analysis (PLS-DA) score plot for discriminating the cell metabolome from Ctrl, LPS, and ACT (50 μM) groups in positive ion mode. **(H)** Heat map of the differential metabolites that were altered in the LPS group compared to the Ctrl group and the ACT group compared to the LPS group (*n* = 6). **(I)** The disturbed metabolic pathways in ACT group.

The transcriptomic analysis was performed by RNA-seq to explore the mechanism of ACT in BV-2 cells from an overall level. PCA illustrated that the control, LPS, and ACT groups could be well distinguished (Figure 3A). It revealed 899 differentially expressed genes (DEGs) between the control group and LPS group, and 49 DEGs between the LPS group and ACT group (Figure 3B). Consistently, GO (Figure 3C) and KEGG enrichment analysis (Figure 3D) uncovered that the effect of ACT was involved in the NF-κB pathway. The set of genes associated to the NF-κB pathway was further confirmed, and their homeostasis was certainly affected by LPS. As expected, ACT significantly affected their expressions (Figure 3E).

The NF-κB pathway is a classical pathway to regulate the progression of inflammation, ultimately resulting in the release of pro-inflammatory factors. To gain mechanistic support, the key protein of the NF-κB pathway was evaluated by Western blot. LPS stimulation led to the activation of NF-κB, associated with promoting M1 polarization. Consistent with RNA-seq analysis, ACT inhibited LPS-stimulated NF-κB phosphorylation (Figure 3F). Therefore, ACT can relieve the LPS-induced M1 polarization *via* the NF-κB pathway in BV-2 cells.

### ACT Impaired Arginine Biosynthesis as Well as Pantothenate and CoA Biosynthesis

Network prediction of ACT revealed that it was related to arginine (Arg) and proline metabolism (Figure 1B). RNA-seq demonstrated that the pathways affected by ACT also included Arg biosynthesis as well as pantothenate and CoA biosynthesis (Figure 3D). It has been suggested that the metabolism disorders of BV-2 cells induced by LPS stimulation is associated with M1 polarization (Orihuela et al., 2016). Thus, untargeted cell metabolome by HPLC-Q-TOF-MS was used to identify the effect of ACT on the cell metabolism. PCA and PLS-DA (Figure 3G) illustrated that the control, LPS, and ACT groups could be well distinguished based on intracellular metabolites. The levels of various metabolites in LPS-induced BV-2 cells were changed after ACT treatment (Figure 3H). Compared with the control group, there were 11 metabolites that changed significantly in the LPS group (Supplementary Table 2), whereas 14 metabolites were distinctly altered after the treatment of ACT (Supplementary Table 3), involving 11 metabolic pathways (Figure 3I). The effect of ACT mainly consisted of regulating amino acid metabolism, nucleotide metabolism, energy metabolism, and metabolism of cofactors and vitamins. Interestingly, the metabolic pathways obtained by metabolome were consistent with network prediction and RNA-seq, including Arg biosynthesis as well as pantothenate and CoA biosynthesis. The above showed that ACT could regulate Arg biosynthesis as well as pantothenate and CoA biosynthesis in LPS-stimulated BV-2 cells.

### ACT Mitigated LPS-Induced BV-2 Mitochondrial Dysfunction

**FIGURE 4:**
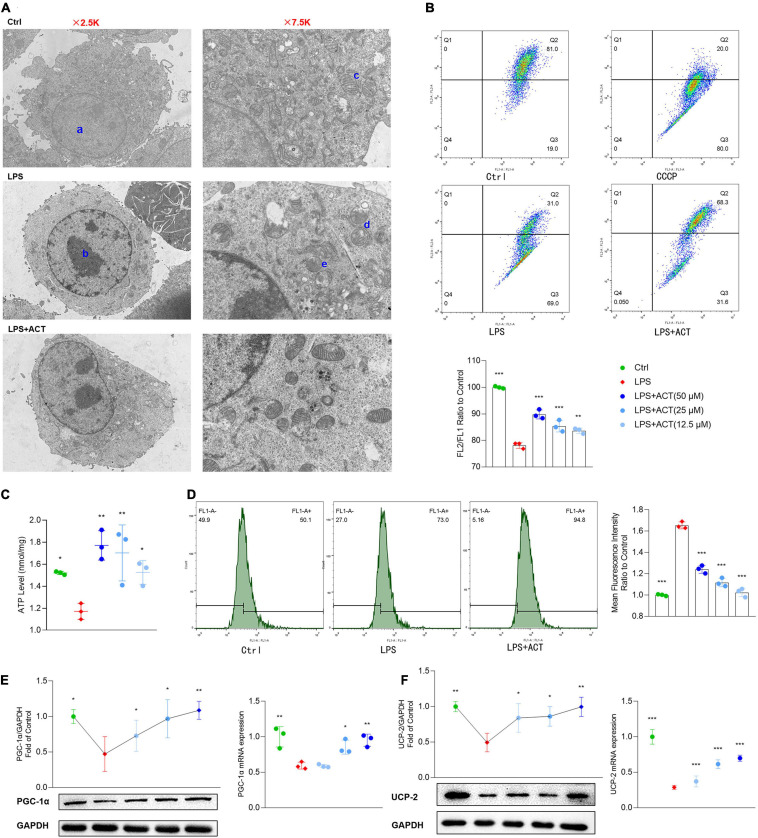
Effects of ACT on mitochondrial function in LPS-treated BV-2 cells. **(A)** Ultrastructural changes in the different groups of BV-2 cells under the transmission electron microscopy. a, normal nucleus. b, nucleus chromatin condensation. c, normal mitochondria. d, mitochondrial cristae disarranged or even disappeared. e, swollen and vacuolated mitochondria. **(B)** The representative images and bar graph of JC-1 red and green fluorescence ratio. **(C)** Bar graph showing quantified statistics of ATP level. **(D)** The representative images of DCFH-DA staining and bar graph of intracellular ROS production. **(E)** ACT distinctly decreases the protein and mRNA levels of PGC-1α. **(F)** ACT distinctly decreases the protein and mRNA levels of UCP-2. *P < 0.05, **P < 0.01, ***P < 0.005, versus the LPS group. *n* = 3.

Mitochondria are at the core of metabolic pathways. Evolving evidence shows that mitochondria are a key player in microglial M1/M2 polarization. An overview of the mitochondria status in morphology and cell distribution were judged by TEM. After LPS stimulation, the chromatin of BV-2 cells was condensed, and the cytoplasm and mitochondria were decreased (Figure 4A). In addition, the mitochondrial cristae of the LPS group were disarranged or even disappeared, exhibiting partial cristolysis, size reduction, and round-shaped morphology. Interestingly, ACT treatment could alleviate LPS-induced morphological changes on mitochondria. By turns, we examined the mitochondria function of LPS-treated BV-2 cells, including MMP and ATP production. The MMP (Figure 4B) and ATP production in mitochondria (Figure 4C) were significantly improved in the ACT group compared to those in the LPS group. Mitochondrial dysfunction may be related to the increased level of ROS in the cell. Flow cytometry analysis revealed that the content of ROS was overloaded in the LPS group (Figure 4D). Fortunately, ACT eliminated excessive ROS. It suggests that ACT may restore mitochondria function by clearing ROS.

### ACT Restored Mitochondria Function Through the Upregulation of PGC-1α and UCP-2

Peroxisome proliferative-activated receptor-γ co-activator-1α (PGC-1α) plays an important role in mitochondrial biogenesis (Bi et al., 2019). The stimulation of LPS decreased the expression of PGC-1α, exhibiting mitochondria dysfunction. Remarkably, PGC-1α gene mRNA and protein expression were significantly reversed by ACT treatment in LPS-treated BV-2 cells (Figure 4E).

The mitochondrial uncoupling protein-2 (UCP-2) was also known to regulate mitochondrial function. As a downstream protein of PGC-1α, it can control LPS-induced MMP depolarization and ROS production. Recent reports indicate that it is central to the process of microglial activation, with opposite regulation of M1 and M2 polarization (De Simone et al., 2015). Western blot results showed that UCP-2 protein level was decreased after LPS stimulation. The co-treatment of LPS and ACT could upregulate the expression of UCP-2 compared to the LPS treatment. The mRNA expression level of UCP-2 also showed similar changes (Figure 4F). To sum up, ACT restored mitochondria function through the upregulation of PGC-1α and UCP-2.

### ACT Repressed Microglia M1 Polarization *via* AMPK Activation

**FIGURE 5:**
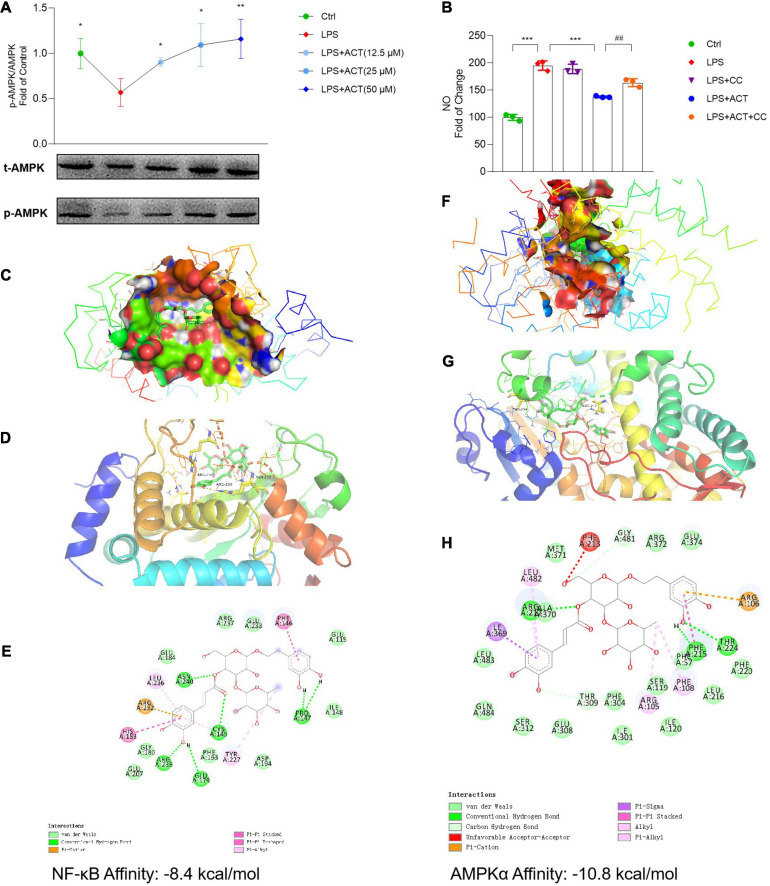
ACT bond to and inhibited NF-κB as well as activated AMPKα. **(A)** ACT distinctly increases the phosphorylation level of AMPKα. **(B)** The effect of compound C (1 μM) on NO level. ***P < 0.001, versus the LPS group. ##*P* < 0.01, versus the LPS+ACT group. *n* = 3. **(C)** A three-dimensional visual of the binding sites between ACT and NF-κB. **(D)** A two-dimensional close-up of the molecule binding pocket from the side. **(E)** Amino acid residues. **(F)** A three-dimensional visual of the binding sites between ACT and AMPKα. **(G)** A two-dimensional close-up of the molecule binding pocket from the side. **(H)** Amino acid residues.

As a key cellular energy sensor, AMP-activated protein kinase (AMPK) plays an important role in maintaining cell metabolism balance. At the same time, PGC-1α is a downstream protein of AMPK. As shown in the analysis of network model, ACT might regulate the AMPK pathway (Figure 1B). Results uncovered that LPS inhibited the activation of AMPK, resulting in cell metabolism disorders and mitochondrial dysfunction. It is noteworthy that ACT could dose-dependently increase the protein expression of p-AMPK (Figure 5A). It is suggested that ACT might increase the expression of PGC-1α and restore mitochondrial function by activating the AMPK signaling pathway.

In this study, to investigate whether the activation of AMPK contributed to the regulation effect of ACT on M1/M2 polarization, compound C (CC) was employed to inhibit the effect of AMPK. In contrast to the downregulated NO level in the ACT treatment group, CC partly blocked the effect of ACT on NO level (Figure 5B). Based on these results, ACT could regulate M1/M2 polarization of BV-2 cells by the activation of AMPK.

### ACT Bond to and Inhibited NF-κB as Well as Activated AMPKα

Molecular docking was applied to confirm whether ACT binds to the NF-κB and AMPKα proteins. Findings demonstrated that the binding energy of ACT and NF-κB was -8.4 kcal/mol, while that of ACT and AMPKα was -10.8 kcal/mol. Significant affinities verified that ACT bound to NF-κB and AMPKα directly (Figures 5C,D,F,G). Subsequently, the possible binding modes and interactions within the amino acid pockets were further explored, including 11 amino acid residues of NF-κB (Figure 5E) as well as 12 amino acid residues of AMPKα (Figure 5H). These results indicated that ACT might directly affect NF-κB and AMPKα to attenuate BV-2 microglia M1 polarization and promote the M2 phenotype.

## Discussion

Alzheimer’s disease is a progressive neuronal and cognitional dysfunction disease with complex dysregulated mechanisms (Fakhri et al., 2020). Accumulating evidence has demonstrated that there is a significant association between microglia-driven inflammation in the brain. Microglia are macrophages in the brain (Afridi et al., 2020) that could be activated into a classical M1 inflammatory phenotype, characterized by enhanced secretion of proinflammatory cytokines (Hanslik and Ulland, 2020). As a consequence, excessive M1 activation could accelerate neuron damage and neurodegeneration, and even exacerbate AD (Bagheri-Mohammadi, 2020). Thus, it is imperative to seek new therapeutic approaches aimed at controlling microglia polarization points, so as to provide adaptive benefits.

Our previous work has verified that ACT has significant effects on improving the learning and memory ability, and protecting the neurons in rats (Chen Y. et al., 2020). Consistently, the present study also proved that ACT could relieve AlCl_3_-induced dyskinesia and cholinergic system disorder in zebrafish. Excitedly, ACT presented remarkable anti-inflammatory activities in LPS-induced BV-2 cells.

ACT suppressed M1 polarization by inhibiting the NK-κB pathway. Besides the NF-κB pathway, RNA-seq and HPLC-Q-TOF-MS analysis also discovered that ACT treatment could affect arginine biosynthesis as well as pantothenate and CoA biosynthesis. It has been widely reported that iNOS could metabolize Arg to NO and citrulline whereas Arg-1 could hydrolyze Arg to ornithine and urea, which are associated with neuron repair (Rath et al., 2014). LPS stimulation led to the upregulation of iNOS (Figure 2D) and downregulation of Arg-1 (Figure 2E), resulting in increased NO level (Figure 2C). The data uncovered that ACT alleviated the increased NO level through arginine biosynthesis.

Pantothenic acid (PA) is the primary substrate for pantothenate kinase (Kumar et al., 2020) and the rate-limiting metabolite in CoA biosynthesis. PA is the obligate precursor of acetyl-CoA, which is of particular importance for cholinergic neurons (Xu et al., 2020) and participates in tricarboxylic acid (TCA) cycle (Atamna, 2004). Recent studies showed that elevated concentration of CoA would lead to altered mitochondrial morphology and lower ATP content (Kumar et al., 2020). LPS-induced BV-2 cells exhibited a decrease in the number of mitochondria and a change of mitochondrial shape. After LPS induction, the production of ROS in BV-2 cells increased. Then, the overladen ROS caused membrane phospholipid to be attacked by free radical (Magnani et al., 2020), leading to the loss of MMP and, in turn, mitochondrial dysfunction and ATP depletion. It was outstanding that ACT treatment mitigated the decrease of MMP and ATP content. These data suggested that ACT induced mitochondrial dysfunction by regulating pantothenate and CoA biosynthesis.

It has been extensively reported that microglia polarization is closely associated with cell metabolism (Orihuela et al., 2016). In particular, as the metabolic hub, mitochondria play remarkable roles in regulating cell metabolism. Recently, mitochondria have been positioned as a key determinant point in microglia polarization (Harry et al., 2020). To better understand the mechanism of ACT, we judged the functional axis of mitochondria by Western blot. It revealed that ACT induced mitochondrial dysfunction by the activation of the AMPKα/PGC-1/UCP-2 axis.

PGC-1α and UCP-2 are both related to mitochondrial biogenesis (Uittenbogaard and Chiaramello, 2014; de Oliveira Bristot et al., 2019), and they can be thought as the master regulators of ROS (Jamwal et al., 2020). Reports indicated that PGC-1α-mediated mitochondrial biogenesis and reduction of ROS are dependent on induction of UCP-2 (Uittenbogaard and Chiaramello, 2014; de Oliveira Bristot et al., 2019; Jamwal et al., 2020). Due to overloading ROS, the expression of PGC-1α and UCP-2 was downregulated in LPS-induced BV-2 cells. It suggested that ACT could eliminate excessive ROS through PGC-1α and UCP-2, thus restoring the mitochondrial function. According to the literature, the alteration of PGC-1α in BV-2 cells could contribute to regulating polarization. Interestingly, previous report has found that increased PGC-1α expression inhibited the NF-κB activity in LPS-induced BV-2 cells (Yang et al., 2017), which qualified the relationship between PGC-1α and NF-κB in our study.

The expression of PGC-1α is affected by upstream pathway proteins, such as AMPK. AMPK is a key protein for the maintenance of cellular homeostasis (Qiu et al., 2020), playing various roles in promoting M2 polarization of microglia (Chu et al., 2019). It modulates metabolic pathways in cells (Szewczuk et al., 2020). We found that ACT promoted the activation of AMPK. At the same time, the application of compound C (AMPK inhibitor) blocked the effect of ACT on attenuating LPS-induced NO excess. Therefore, ACT also suppressed LPS-stimulated M1 polarization *via* the AMPK signaling pathway.

**FIGURE 6:**
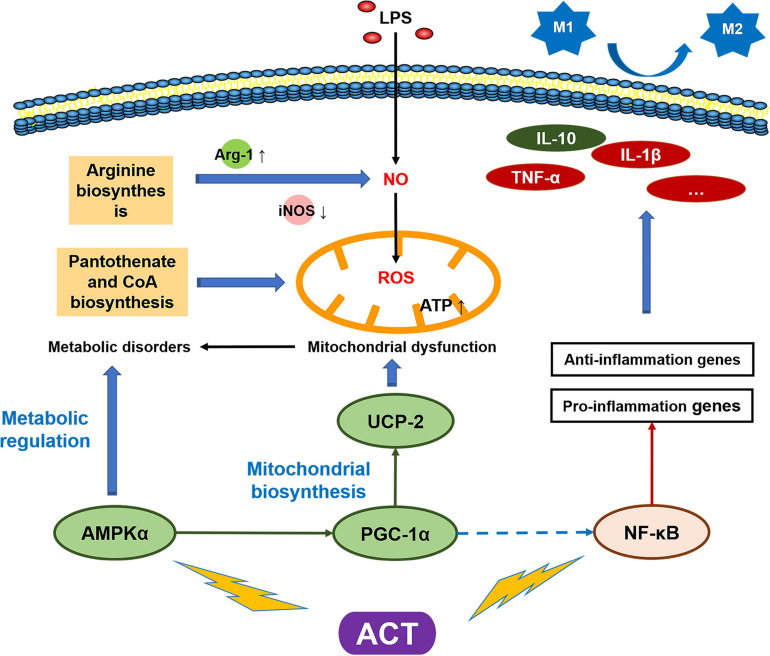
Schematic model of the mechanism by which ACT suppresses LPS-induced M1 polarization *via* regulating AMPK and NF-κB signaling pathways.

It is the first time to report the mechanism of ACT on regulating microglia polarization (Figure 6). The data supported that ACT could be developed as a therapeutic agent for neurodegenerative disease associated with neuroinflammation, such as AD. In particular, we linked the microglia polarization with cell metabolism, explaining the effect of ACT through the alteration of mitochondria function. The identification of this metabolic axis, as a target of a unique entity, may lead to much better therapeutic approaches against microglia M1 polarization, particularly in AD.

## Data Availability Statement

The datasets presented in this study can be found in online repositories. The names of the repository/repositories and accession number(s) can be found below: The RNA-seq data was downloaded from the NCBI database (https://www.ncbi.nlm.nih.gov) under the accession number PRJNA679710.

## Ethics Statement

The animal study was reviewed and approved by the Animal Ethics Committee of China Pharmaceutical University.

## Author Contributions

Y-QL, YC, Y-YS, and S-QJ performed conceptualization and methodology. Y-QL performed sample preparation. Y-QL, S-QJ, Y-YS, and S-SW performed data curation and experimental work. Y-QL, YC, and X-LJ performed writing and original draft preparation. FL and PL performed supervision, reviewing, and editing. All authors contributed to the article and approved the submitted version.

## Conflict of Interest

The authors declare that the research was conducted in the absence of any commercial or financial relationships that could be construed as a potential conflict of interest.

## Footnotes

1 https://www.genecards.org

2 http://sea.bkslab.org

3 http://www.swisstargetprediction.ch

4 http://www.lilab-ecust.cn/pharmmapper

5 https://www.disgenet.org/

6 https://string-db.org

7 https://david.ncifcrf.gov

8 https://www.metaboanalyst.ca

9 https://hmdb.ca

10 www.majorbio.com

11 http://deweylab.biostat.wisc.edu/rsem

12 https://www.rcsb.org
