# Supplementary material for: An Inhibitor of NF-κB and an Agonist of AMPK: Network Prediction and Multi-Omics Integration to Derive Signaling Pathways for Acteoside Against Alzheimer’s Disease
Source: Front Cell Dev Biol. 2021 Jul 19;9:652310. doi: 10.3389/fcell.2021.652310 (PMC8327963; doi:10.3389/fcell.2021.652310)
Supplement: Supplementary file 1 [file Table_1.DOC]

**Table S1. PCR primers for mRNA expression detection.**

| **Gene symbol** | **Forward primer (5’-3’)** | **Reverse primer (3’-5’)** |
| --- | --- | --- |
| β-actin | GTGACGTTGACATCCGTAAAGA | GCCGGACTCATCGTACTCC |
| IL-1β | GACAAAATACCTGTGGCCTTG | GATGTACCAGTTGGGGAACTCT |
| TNF-α | TGAGCACAGAAAGCATGATC | TACAGGCTTGTCACTCGAATT |
| iNOS | CAAGCACATTTGGGAATGGAGA | CAGAACTGAGGGTACATGCTGGAG |
| IL-10 | CATTCCCAGAGGAATTGCAT | CCAGGGAGATCCTTTGATGA |
| CD86 | CAACGGAATTAGGAAGAC | CTCTGTATGCAAGTTTCC |
| CD206 | CAAGGAAGGTTGGCATTTGT | CCTTTCAGTCCTTTGCAAGC |
| Arg-1 | TCACCTGAGCTTTGATGTCG | CTGAAAGGAGCCCTGTCTTG |
| TGF-β | TGGCGTTACCTTGGTAACC | GGTGTTGAGCCCTTTCCAG |
| PGC-1α | TGATGTGAATGACTTGGATACAGACA | GCTCATTGTTGTACTGGTTGGATATG |
| UCP-2 | GCGTTCTGGGTACCATCCTA | GCTCTGAGCCCTTGGTGTAG |
