# Supplementary material for: An Inhibitor of NF-κB and an Agonist of AMPK: Network Prediction and Multi-Omics Integration to Derive Signaling Pathways for Acteoside Against Alzheimer’s Disease
Source: Front Cell Dev Biol. 2021 Jul 19;9:652310. doi: 10.3389/fcell.2021.652310 (PMC8327963; doi:10.3389/fcell.2021.652310)
Supplement: Supplementary file 2 [file Table_2.DOC]

**Table S2. The differential metabolites identified by comparison of Ctrl and LPS group in BV-2 cells.**

| **No.** | **Rt (min)** | **HMDB_ID** | **Formula** | **Identification** | **mz** | **FDR** | **VIP** | **ESI mode** |
| --- | --- | --- | --- | --- | --- | --- | --- | --- |
| 1 | 0.6392 | HMDB0028783 | C_8_H_14_N_2_O_3_S | Cysteinyl-Proline | 219.0798 | 0.040806 | 1.191 | + |
| 2 | 0.6417 | HMDB0000235 | C_12_H_17_N_4_OS | Thiamine | 265.1123 | 0.022722 | 2.2719 | + |
| 3 | 0.8191 | HMDB0000517 | C_6_H_14_N_4_O_2_ | L-Arginine | 175.1189 | 0.022179 | 2.6729 | + |
| 4 | 0.9179 | HMDB0000158 | C_9_H_11_NO_3_ | L-Tyrosine | 182.0812 | 0.028899 | 2.1687 | + |
| 5 | 1.0419 | HMDB0028717 | C_11_H_21_N_5_O_3_ | Arginylproline | 294.1537 | 0.004215 | 2.9455 | + |
| 6 | 1.0584 | HMDB0034301 | C_5_H_11_N | Piperidine | 86.0964 | 0.01583 | 3.7697 | + |
| 7 | 1.5843 | HMDB0000159 | C_9_H_11_NO_2_ | L-Phenylalanine | 166.0863 | 0.022179 | 4.159 | + |
| 8 | 1.6581 | HMDB0006804 | C_3_H_2_O_2_ | Propynoic acid | 115.0037 | 1.96E-05 | 1.6344 | - |
| 9 | 3.076 | HMDB0001173 | C_11_H_15_N_5_O_3_S | 5'-Methylthioadenosine | 298.0968 | 0.039998 | 3.4131 | + |
| 10 | 6.9615 | HMDB0030244 | C_17_H_13_NO_3_ | Graveolinine | 302.0788 | 0.025397 | 3.4131 | + |
| 11 | 11.8 | HMDB0031797 | C_11_H_15_NO_2_ | 2-Isopropylphenyl methylcarbamate | 194.1176 | 0.022179 | 2.0951 | + |
