# Supplementary material for: An Inhibitor of NF-κB and an Agonist of AMPK: Network Prediction and Multi-Omics Integration to Derive Signaling Pathways for Acteoside Against Alzheimer’s Disease
Source: Front Cell Dev Biol. 2021 Jul 19;9:652310. doi: 10.3389/fcell.2021.652310 (PMC8327963; doi:10.3389/fcell.2021.652310)
Supplement: Supplementary file 3 [file Table_3.DOC]

**Table S3. The differential metabolites identified by comparison of LPS and ACT group in BV-2 cells.**

| **No.** | **Rt (min)** | **HMDB_ID** | **Formula** | **Identification** | **mz** | **FDR** | **VIP** | **ESI mode** |
| --- | --- | --- | --- | --- | --- | --- | --- | --- |
| 1 | 0.6952 | HMDB0000641 | C_5_H_10_N_2_O_3_ | L-Glutamine | 169.0584 | 0.000226 | 2.6852 | + |
| 2 | 0.7607 | HMDB0014467 | C_9_H_11_FN_2_O_5_ | Floxuridine | 245.0579 | 0.000831 | 2.0959 | - |
| 3 | 0.7897 | HMDB0011617 | C_10_H_14_N_5_O_7_P | Adenosine 2'-phosphate | 370.0523 | 0.011154 | 2.7231 | + |
| 4 | 0.8324 | HMDB0006899 | C_5_H_10_N_2_O_3_ | Alanylglycine | 147.0764 | 0.041506 | 2.9497 | + |
| 5 | 0.8353 | HMDB0031861 | C_6_H_6_N_2_O | 2-Acetylpyrazine | 123.0553 | 0.005876 | 2.9389 | + |
| 6 | 0.8704 | HMDB0033951 | C_5_H_11_NO_2_S | Racemethionine | 150.0583 | 0.000727 | 2.807 | + |
| 7 | 0.8949 | HMDB0001397 | C_10_H_14_N_5_O_8_P | Guanosine monophosphate | 364.0653 | 0.022102 | 3.1333 | + |
| 8 | 1.0419 | HMDB0028717 | C_11_H_21_N_5_O_3_ | Arginylproline | 294.1537 | 0.041506 | 3.2535 | + |
| 9 | 2.9236 | HMDB0000159 | C_9_H_11_NO_2_ | L-Phenylalanine | 188.0682 | 0.012582 | 2.0327 | + |
| 10 | 6.3388 | HMDB0029336 | C_36_H_49_N_5_O_6_ | Nummularine A | 648.3756 | 0.005553 | 2.428 | + |
| 11 | 10.1396 | HMDB0000210 | C9H17NO5 | Pantothenic acid | 220.118 | 0.011785 | 1.3618 | - |
| 12 | 11.8337 | HMDB0000501 | C_27_H_44_O_2_ | 7-Ketocholesterol | 423.3233 | 0.001867 | 2.0349 | + |
| 13 | 15.343 | HMDB0006285 | C_20_H_26_O_3_ | 4-oxo-Retinoic acid | 313.1809 | 0.010718 | 1.5875 | - |
